# Comparison of Habitat Suitability Models for *Haemaphysalis longicornis* Neumann in North America to Determine Its Potential Geographic Range

**DOI:** 10.3390/ijerph17218285

**Published:** 2020-11-09

**Authors:** Jamyang Namgyal, Isabelle Couloigner, Tim J. Lysyk, Shaun J. Dergousoff, Susan C. Cork

**Affiliations:** 1 Department of Ecosystem and Public Health, Faculty of Veterinary Medicine, University of Calgary, Calgary, AB T2N 1N4, Canada; icouloig@ucalgary.ca (I.C.); timlysyk@gmail.com (T.J.L.); sccork@ucalgary.ca (S.C.C.); 2 Department of Geography, University of Calgary, Calgary, AB T2N 1N4, Canada; 3 Agriculture and Agri-Food Canada, Lethbridge Research and Development Center, Lethbridge, AB T1J 4B1, Canada; shaun.dergousoff@canada.ca

**Keywords:** *Haemaphysalis longicornis*, Asian longhorned tick, MaxEnt, North America

## Abstract

*Haemaphysalis longicornis* Neumann, 1901 is a vector of many pathogens of public and veterinary health importance in its native range in East Asia and introduced range in Oceania. In North America, this tick was first detected in New Jersey in 2017. Currently, this tick has been reported from 15 states of the United States. In this study, we modeled the habitat suitability of *H. longicornis* using the MaxEnt modeling approach. We separated occurrence records from the published literature from four different geographical regions in the world and developed MaxEnt models using relevant environmental variables to describe the potential habitat suitability of this tick in North America. The predictive accuracy of the models was assessed using the U.S. county locations where this tick species has been reported. Our best model predicted that the most suitable North American areas for geographic expansion of *H. longicornis* are from Arkansas–South Carolina to the south of Quebec–Nova Scotia in the east, and from California to the coast of British Columbia in the west. Enhanced surveillance and further investigation are required to gain a better understanding of the role that this tick might play in the transmission of diseases to humans and animals in North America.

## 1. Introduction

*Haemaphysalis longicornis* Neumann, 1901 (Acari, Ixodidae), the Asian longhorned tick, is a three-host, tropical tick whose native range includes Japan, China, eastern Russia, and Korea [1]. It has also become established in Australia, New Zealand, and the western Pacific islands (New Caledonia, Fiji, Western Samoa, Tonga, Vanuatu) [1,2,3]. In East Asia, *H. longicornis* is the main vector for transmission of severe fever with thrombocytopenia syndrome virus (SFTSV) in humans [4]. This emerging zoonotic disease caused by a novel bunyavirus was first identified in China in 2009 [5] and then in South Korea and Japan in 2013 [6,7]. Genetically, SFTSV is closely related to the heartland virus (HRTV), which has been isolated in the United States [8,9,10]. In Japan, *H. longicornis* is also considered a vector of *Rickettsia japonica*, which causes Japanese spotted fever in humans [11]. In Australia and New Zealand, this tick species is a vector for the protozoan parasite *Theileria orientalis* Ikeda that causes bovine theileriosis [3,12,13]. Theileriosis can lead to severe and life-threatening anemia in cattle [3]*. Haemaphysalis longicornis* is also a competent vector for other bovine disease organisms such as *Babesia ovata*, *B. major*, and *Anaplasma bovis* in New Zealand [13], and the DNA of *Ehrlichia* and *Borrelia* spp. has been detected in ticks collected from its native range in East Asia [14,15].

In North America, *H. longicornis* was first detected on a sheep in New Jersey in August 2017 [16]. Retrospective investigations have revealed that this tick has actually been present in the United States since 2010 [17], but it was initially misidentified as the native rabbit tick, *Haemaphysalis leporispalustris* (Packard, 1869) [18]. Since then, this tick has been reported in 118 counties in 15 states of the United States [19]. So far, there has not been any detection of this tick in Canada [20,21]. To our knowledge, there has not been any human pathogen detected in field-collected *H. longicornis* in the United States; however, there is a concern that this tick has the potential to transmit endemic pathogens such as *Anaplasma*, *Babesia*, and *Rickettsia* species [20]. The first human bite case by *H. longicornis* tick in the United States was reported from New York state in 2018 [22]. This tick species has parthenogenetic, bisexual, and aneuploid populations [23]. The parthenogenetic population is capable of asexual reproduction, whereby females can lay eggs without fertilization by the males [21]. In New Jersey, among more than the 1100 ticks collected from the index site, only one male specimen was found, indicating that the invasive population was parthenogenetic [16]. This was supported by a genetic analysis, which indicated *H. longicornis* ticks in the United States are more similar to those of parthenogenetic populations than those of bisexual populations [24]. The parthenogenetic populations are distributed in Australia, New Zealand, New Caledonia, Fiji, New Hebrides, Tonga, northeastern Russia, northern Japan, Kyushu and Yakushima in southern Japan, and Sichuan and Shanghai in China [1,25,26]. Modeling has indicated areas along the Gulf and Atlantic coast of eastern North America as the potential geographical range of *H. longicornis* expansion [27]. Other modeling studies have indicated the potential expansion and distribution of this tick species in eastern North America from southern Canada to the Gulf coast, and in a small temperate area on the west coast [28], and the southeastern United States, the Pacific Northwest, and central and southern Mexico [29].

In this study, we used the *H. longicornis* presence data from Rochlin (2018) [28] augmented with data from Zhang et al. (2019) [30] with MaxEnt [31] to model the habitat suitability of *H. longicornis* in North America. We separated the *H. longicornis* presence data into native range and introduced range and then built MaxEnt models for each of them to compare the habitat suitability predictions for North America. The objectives of this study were to (1) separate the global *H. longicornis* occurrence data for different regions in the world and build competing models using environmental predictors identified by Rochlin (2018) [28], (2) build competing models for different regions using environmental variables from WorldClim [32] and ENVIREM [33], and (3) compare habitat suitability predictions for North America by these competing models and subsequently select the two best models to describe the potential distribution and expansion of *H. longicornis* in North America. The findings from this study will help develop cost-effective surveillance programs, targeting areas within the predicted range of *H. longicornis* occurrence under the current environment.

## 2. Materials and Methods

We used the *H. longicornis* presence data containing 261 occurrence points from Rochlin (2018) [28] and 146 occurrence points at China’s county-level from Zhang et al. (2019) [30]. The environmental predictors used were bio 1 (annual mean temperature), bio 5 (maximum temperature of the warmest month), and bio 12 (annual precipitation) downloaded from WorldClim (https://www.worldclim.org/) [32] at 2.5 min spatial resolution and Global Ecological Zone (GEZ) (http://www.fao.org/geonetwork/srv/en/main.home#ecology) based on Rochlin (2018) [28]. GEZ was rasterized at the same 2.5 min spatial resolution. The other 12 WorldClim variables and 3 of the ENVIREM [33] variables, i.e., annual potential evapotranspiration (annualPET), Thornthwaite aridity index, and continentality, were also considered at 2.5 min resolution. For North America, 97 occurrence records were obtained by georeferencing the coordinates of the centroids of the counties that have reported the presence of *H. longicornis* in the United States as of April 2020 [19]. Data preparation was done using ArcGIS^®^ (v 10.6.1) software by ESRI (Toronto, Canada) and R (R Core Team, Vienna, Austria) [34] with the *raster* [35] and *rgdal* packages [36].

As the occurrence records were of presence-only data, maximum entropy distribution modeling, or MaxEnt modeling was used to create habitat suitability maps of *H. longicornis* in North America. Statistical modeling was done by running MaxEnt (New York, U.S.A.) [31] in R [34] within *Dismo* [37], *MIAmaxent* [38], and *ENMeval* [39] packages. The raster stack containing the environmental predictors was separated into four geographic areas of interest: (1) the current range of *H. longicornis* in both East Asia and Oceania (entire distribution), (2) the native range of *H. longicornis* in East Asia (East Asia), (3) the introduced range of *H. longicornis* in Oceania (Oceania), and (4) the parthenogenetic range of *H. longicornis* in East Asia (native parthenogenetic). The approximate occurrence locations where parthenogenetic populations are reported in the literature [1,25,26] were delineated from the native range and used as the parthenogenetic range data. For each area of interest, the environmental values were extracted at the occurrence locations. Random background points (around 1000) were generated from the area of unsuitable habitat modeled from the “BIOCLIM” algorithm (a classic presence-only climate envelope model) [37] for each region to be used as pseudo-absence points [40], and their corresponding environmental values were extracted. The environmental data for all four areas of interest were compared using box plots. The frequency of observed presence (FOP) plots [38] for the predictors of interest were also analyzed to determine whether the patterns of occurrence specific to the study area were compatible with the ecological knowledge of the *H. longicornis* (Figure S1).

We used a two-step strategy to predict the habitat suitability of *H. longicornis* in North America. For the first modeling step, we used the same environmental predictors as Rochlin (2018) [28], i.e., bio 1, bio 5, bio 12, and GEZ, to develop a series of candidate MaxEnt models (Table S1) with a variety of settings. Random 5-fold partitioning of the presence and absence data (80% training, 20% testing) was used to assess each area of interest to find the best model based primarily on the corrected Akaike Information Criteria (AICc) [41,42]. The modeled relationships of the original predictor variables (i.e., the “feature classes” and “regularization multiplier”) for each best MaxEnt model are presented in Table S1. The contribution and permutation importance of the environmental variables of the best models generated for each area of interest were assessed from the MaxEnt output. These models were then projected into North America to identify areas of greatest predicted habitat suitability. The predicted maps of the final models were compared for niche similarity [43], and the predictive accuracy of the models was ranked using the known presence locations in the United States based on both the correlation of the predicted and observed data and the AICc [44]. Further, the *H. longicornis* habitat suitability in North America was mapped into 5 classes (0–0.2, very low; 0.2–0.4, low; 0.4–0.6, moderate; 0.6–0.8, high; 0.8–1.0, very high) following the classification of Zuliani et al. [44].

For the second modeling step, we used 12 bioclimatic variables from WorldClim Version 2 [32] and 3 variables (i.e., annualPET, Thornthwaite aridity index, and continentality) from ENVIREM [33]. These variables were used to find the best subsets of predictors for MaxEnt modeling using a forward stepwise selection process [38]. Using the subsets of predictors identified, a series of candidate MaxEnt models (Table S2) were developed using the same methodology described for the first modeling step. Then, the two best MaxEnt models from the second step were identified using both correlation and AICc to predict habitat suitability for the *H. longicornis* in North America.

## 3. Results

**Figure 1:**
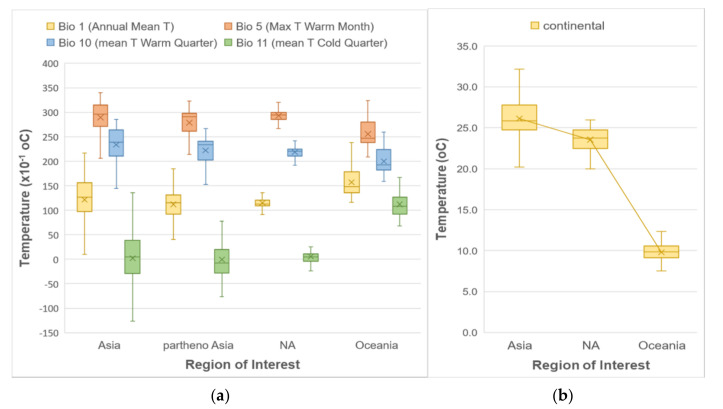
Boxplots of different temperature predictors, (**a**) WorldClim and (**b**) ENVIREM, showing the distribution of their values at the *H. longicornis* presence locations according to the geographic region.

For the first step of our analysis, we looked at whether using all occurrences from the entire known geographic distribution of *H. longicornis*, occurrences from only its native range, as well as its native parthenogenetic range, or occurrences from only its introduced range would make a difference in determining its habitat suitability prediction in North America as the analysis of the climatic predictors showed some differences and similarities depending on regions of interest (Figure 1). Examination of the boxplots in Figure 1 showed that predictors for the North America region follow a similar temperature pattern as the native and parthenogenetic range in East Asia (both for means—represented by the crosses in box plots—and medians) to a greater extent than the ones from the introduced range in Oceania. Precipitation patterns did not vary much among the different regions, and hence they are not included in this figure.

Four competing MaxEnt models were generated based on the environmental predictors from Rochlin [28]. The relative importance of the environmental predictors on the models generated was assessed using MaxEnt’s permutation importance for each model (Table 1). The best features (linear–quadratic (LQ)) and their corresponding beta-multipliers (rm) were selected (Table S1). The relative importance of the environmental predictors varied depending on the geographic region. For the entire distribution range and East Asia range, the two most important predictors were bio 1 (annual mean temperature) at 77.6% and 41.2%, respectively, followed by GEZ—global ecozones—at 10.3% and 33.6%, respectively. For Oceania, the most important were bio 5 (max temperature of warmest month) at 74.8%, followed by bio 12 (annual precipitation) at 13.7%; for the native parthenogenetic range, it was bio 1 at 51.48%, followed by GEZ at 26.54%.

**Figure 2:**
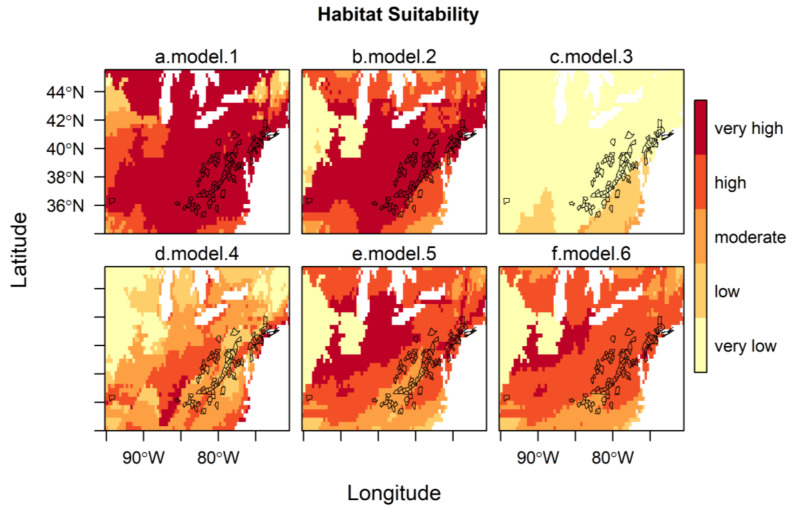
Habitat suitability of *H. longicornis* under current climatic and environmental conditions in the eastern United States, with outlines of U.S. counties with reported *H. longicornis* occurrences [19]. The habitat suitability of all models is represented by 5 classes (0–0.2, very low; 0.2–0.4, low; 0.4–0.6, moderate; 0.6–0.8, high; 0.8–1.0, very high) following the classification of Zuliani et al. [44]. Models with bio 1, bio 5, bio 12, and (Global Ecological Zone) GEZ using *H. longicornis* occurrence locations from (**a**) the entire range, (**b**) the native range, (**c**) Oceania, (**d**) parthenogenetic range; and (**e**) model with bio 1, GEZ, continentality and f. model with bio 10, bio 11, and GEZ using *H. longicornis* occurrence locations from the native range.

These models were projected onto North America to indicate regions of predicted habitat suitability for *H. longicornis*, and the predictions were assessed based on AUC, correlation, and AICc, using the known occurrences of *H. longicornis* in the eastern United States (Table 2). The four corresponding maps of the habitat suitability for the eastern United States, along with the counties with reported *H. longicornis* occurrences, are shown in Figure 2a–d. The maps show the changes in the level of suitability according to the zone of influence chosen for developing the models, i.e., ranging from very high suitability almost everywhere (model 1) to almost all unsuitable (model 3). All models present good predictability since their AUC values are between 0.83 and 0.97, with a preference for models developed on the entire range and East Asia (AUC > 0.95). However, the model developed on the native zone of occurrences (East Asia) was the best model to predict the habitat suitability of *H. longicornis* in North America, as shown by both correlation (0.68) and AICc.

For the second step of our analysis, we looked at whether using different climatic predictors would improve the predictability of MaxEnt modeling for North America. Only the two best models, i.e., the models applied to the East Asia zone (models 5 and 6), are presented in this paper. The results of the predictor selection are presented in Table S2. Table 3 displays the relative importance of the environmental predictors to each model, while Table 2 presents the assessment of the predictability of each model in North America.

For model 5, GEZ was the most important predictor at 45.4%, followed by bio 1 (annual mean temperature) at 27.7% and continentality (the difference between the mean temperature of warmest month and the mean temperature of the coldest month) at 26.8%, showing that bio 1 and continentality had a similar importance to the model. For model 6, bio 11 (mean temperature of coldest quarter) at 46.4% was the most important predictor, followed by GEZ at 43.0% and bio 10 (mean temperature of warmest quarter) at 10.6%. Both models, as shown in Table 2, had good predictability with an AUC greater than 0.95. The best model of the two is model 6, considering both the correlation (0.64) and AICc (2418.7) metrics. Figure 2d–e presents the corresponding two maps of the habitat suitability for the eastern United States, along with the U.S. counties with reported *H. longicornis* occurrences. It shows that the level of suitability changes according to the model chosen, from high (model 6) to a mixture of moderate and high suitability (model 5). When comparing these two models and the four preceding models from the first modeling step, model 2 was the best model based on both correlation and AICc metrics.

**Figure 3:**
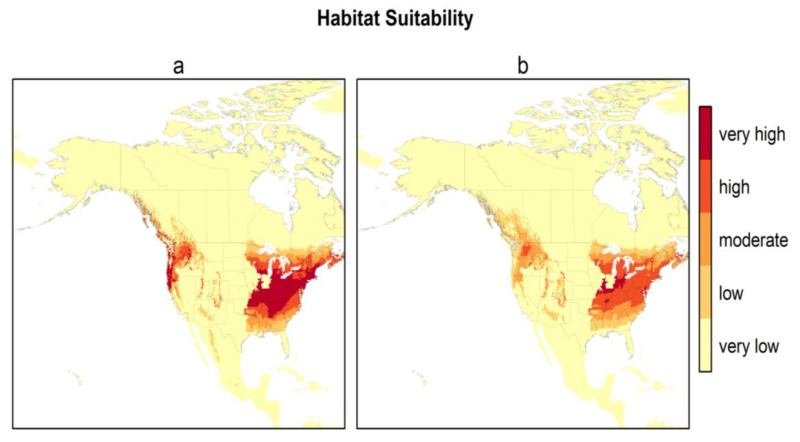
Habitat suitability of *H. longicornis* under current climatic and environmental conditions for North America for the two best models ((**a**) model 2—bio 1, bio 5, bio 12, and (Global Ecological Zone) GEZ using *H. longicornis* occurrence locations from the native range, Asia; (**b**) model 6—bio 10, bio 11, and GEZ using *H. longicornis* occurrence locations from the native range, Asia).

**Figure 4:**
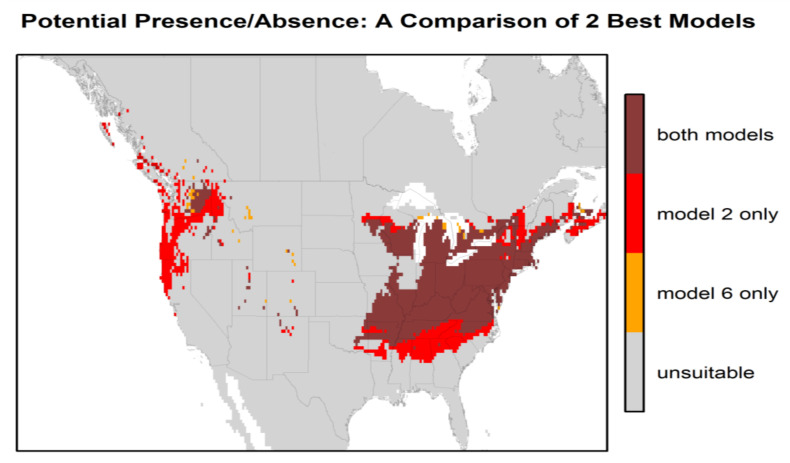
Comparison of the habitat suitability of *H. longicornis* in North America between the 2 best models (model 2 (bio 1, bio 5, bio 12, and GEZ) and model 6 (bio 10, bio 11, and GEZ) for the high suitability probabilities (≥ 0.6).

Figure 3 presents the habitat suitability maps for North America from the two best models (models 2 and 6). Based on these maps, the most suitable areas in North America are found within the temperate zones, i.e., the east, and a narrower area in the west between the Rocky Mountains and the Pacific coast. The difference in North American habitat suitability prediction by the 2 best models is illustrated in Figure 4. The east zone goes from Arkansas–South Carolina to the south of Quebec–Nova Scotia for model 2 and from Tennessee–North Carolina to New York–south of Maine for model 6; the west zone goes from California to the coast of British Columbia for model 2 and encompasses just a small zone east of Washington State for model 6. There is an overlap of 88.4% between these two maps. Model 2 predicts a greater area of “very high” suitability habitat (*p* > 0.8), while Model 6 predicts more areas as “high” suitability habitat (0.6 < *p* < 0.8) for the same area in the east.

## 4. Discussion

The establishment and potential expansion of *H. longicornis* in North America has been of public health and veterinary concern, particularly in the United States and Canada. Globally, this tick species is associated with at least 59 pathogens, of which 30 are potentially pathogenic to humans [45]. The most noted human pathogen transmitted by the *H. longicornis* in its native range in East Asia is the SFTSV [4]. Since its discovery in 2009, cases of SFTSV have been increasingly reported in East Asia [46]. In November 2019, the first case of SFTSV was reported from Taiwan [47], and cases have also occurred in Vietnam [48], suggesting that the SFTSV is expanding its range in Asia. Since *H. longicornis* plays an important role in maintaining and transmitting SFTSV, the possibility of this disease in North America should not be neglected.

To our knowledge, there has been no evidence of any human pathogen transmitted by *H. longicornis* in North America [22,49]. However, there is a concern that this tick might be capable of transmitting *Rickettsia rickettsii,* which causes Rocky Mountain spotted fever. Under laboratory conditions, *H. longicornis* larvae and nymphs, from a colony derived from females collected in New York, were able to acquire and transmit this pathogen [50], further increasing the public health concern. Recently, *Theileria orientalis* Ikeda, a protozoan parasite transmitted by *H. longicornis* in East Asia, New Zealand, and Australia, has been detected in cattle in Virginia, and this has prompted further concerns that this tick species might play a role in the continued transmission of the pathogen causing *Theileria*-associated bovine infectious anemia [17]. In Australia, it is estimated that *T. orientalis* infection has been associated with a loss of AUD 19.6 million per annum for the red meat industry [51]. Therefore, the potential role of this tick in transmitting these pathogens in humans and animals in North America cannot be ignored.

Currently, all published models of *H. longicornis* distribution use climatic variables to predict distribution [27,28,29,45]. Ecological zones have also been included as these can represent a complex of interacting abiotic and biotic variables [28]. The distribution of potential host species was not considered in our work as this is unlikely to limit the distribution of this tick. All stages have been found on a variety of domestic animals including cattle, horses, and dogs [3]. Moreover, all stages of this tick have been collected from white-tailed deer, Odocoileus virginianus *(*Zimmermann, 1780)*,* a widely distributed wildlife species [52], and a variety of other wildlife in the United States [53]. The wide host range of *H. longicornis* may facilitate dispersion over short and long distances. Globally, 77 species of animals are hosts for this tick species [45]. In the United States, the *H. longicornis* has been isolated from 21 species of domestic and wild animals and also from humans [19]. Migratory birds might play an important role in the dispersal of this tick to a new area [29,45]. Further, the parthenogenetic ability of *H. longicornis* is particularly concerning in the context of its potential expansion [29]. A single engorged female can reproduce without mating and establish a population in a new area with suitable environmental conditions [3].

Temperature and precipitation are the most important climatic factors that influence the distribution of *H. longicornis* in both the native (East Asia) and the introduced (Oceania) regions [54,55]. Annual mean temperature greater than 12 °C, mean coldest monthly temperature less than 2 °C, and annual rainfall above 1000 mm are considered to be optimum for *H. longicornis* range expansion in New Zealand [54]. *H. longicornis* tolerates a wide range of temperatures (−2 to 40 °C), but the warm and moist temperate conditions are known to be preferred [3]. Humidity is the limiting factor for the establishment of *H. longicornis* populations, as the threshold for survival and host-seeking activity is 85% relative humidity [3,45]. In our models (models 2 and 6), the most important environmental variables influencing the distribution of *H. longicornis* were bio 1 (annual mean temperature) and GEZ for model 2, and GEZ and bio 11 (mean temperature of coldest quarter) for model 6. In both models, these variables explained more than 70% of the contribution to the model. Our finding that the bio 1 and GEZ are important variables for *H. longicornis* expansion corroborates the findings from the previously published MaxEnt modeling studies [28,45].

We also analyzed the patterns of temperature and precipitation variables in different regions where *H. longicornis* is found. There was a similar pattern of temperature between North America and East Asia, and this might explain why *H. longicornis* habitat suitability in North America was better predicted by the model developed with data from its native range of East Asia alone. A separate model for parthenogenetic *H. longicornis* within its native range was also developed [1,3]. This was done mainly in an attempt to improve the predictive accuracy in North America, as the *H. longicornis* population in North America is also parthenogenetic. We assumed that the parthenogenetic populations might have a different geographical range with different environmental requirements than bisexual populations, which (if true) can improve the predictive accuracy of the models. However, this process did not improve the predictive accuracy of the model, probably because relatively few occurrence records of parthenogenetic *H. longicornis* within its native range were available, and their distribution overlaps that of the bisexual populations.

In our study, we predicted that the most suitable areas for the *H. longicornis* in North America were found within the east zone, i.e., from Arkansas–South Carolina to south of Quebec–Nova Scotia for model 2 and from Tennessee–North Carolina to New York–south of Maine for model 6. In the west zone, the most potentially suitable areas were from California to the coast of British Columbia for model 2 and just a small zone east of Washington State for model 6. The findings are largely in agreement with previous studies that have used the MaxEnt approach [28,29]. However, unlike the predicted distribution from Raghavan et al. (2019) [29], central and southern Mexico were not predicted to be highly suitable areas in our models. This difference in potential prediction could have been due to using a different number of occurrence points and locations or the use of different environmental variables for model calibration. The other MaxEnt modeling study by Zhao et al. (2020) [45], conducted at the global scale, predicted the western coast to be more suitable than the eastern coast in the United States for *H. longicornis*. The use of a relatively low number of occurrence points (249 points) by Zhao et al. (2020) [45] could have affected the predictive accuracy of the models. Further, discrepancies in results among different MaxEnt modeling studies could also occur as a result of using different sources of occurrence data and model settings (i.e., features and regularization in MaxEnt). In our study, the accuracy of habitat suitability predictions for North America was assessed using the 97 known occurrences from the U.S. counties (Figure 2). All 97 locations that have reported the presence of the *H. longicornis* corresponded to the areas predicted as a “very high” suitability by model 2 and predominantly “high” suitability areas by model 6. Most of the potentially suitable areas in the east and west zones correspond to the humid temperate zone and coastal areas, respectively, where humidity may not be the limiting factor.

Reliable presence data is critical for the predictive accuracy of species distribution models. For instance, there was no accurate description of the *H. longicornis* range for parthenogenetic and bisexual populations in the literature; however, we attempted to delineate these ranges and looked at the predictive response of the models. Further, the 97 location records of *H. longicornis* occurrences in the United States were derived from the centroids of positive counties. More accurate publicly available presence and absence data would improve the predictive accuracy of the models. This would facilitate a more cost-effective targeted surveillance for early detection and subsequent tick control response. As *H. longicornis* is a threat to human and animal health, there is a need to embrace a one health approach for its monitoring and control by ensuring timely data sharing and engaging interdisciplinary expertise.

## 5. Conclusions

*Haemaphysalis longicornis* is currently distributed in 118 counties in 15 states of the United States [19]. Previously, specimens of *H. longicornis* were misidentified as the native rabbit tick, *Haemaphysalis leporispalustris* [18], resulting in a delay in an appropriate response to the incursion of this exotic tick. *Haemaphysalis longicornis* has most likely come to North America from East Asia [24]. Companion animals (particularly dogs) entering the United States are thought to be the source [24]. Based on our habitat suitability models, the geographic distribution of the *H. longicornis* will likely continue to expand in North America. Due to the ability of this tick to transmit pathogens, the potential threat of this tick to public and veterinary health should not be ignored. Enhanced tick surveillance to determine the expanding geographical distribution of the *H. longicornis* in North America should be continued. There is also a need for human and animal health monitoring systems to work together to determine the potential role this tick might play in the transmission of diseases to humans and animals in North America. Effective control methods for this tick in North America should be determined using a collaborative one health approach.

## Figures and Tables

**Table 1:** Permutation importance of the environmental predictors applied by Rochlin (2018) [28] in competitive MaxEnt models, as applied to the geographic areas of interest of *H. longicornis* occurrences.

Model	Zone of Influence	GEZ	bio 1	bio 12	bio 5	Features (rm)
1	Entire distribution	10.34	77.63	9.62	2.40	LQ (4.0)
2	East Asia	33.60	41.20	13.46	11.74	LQ (0.5)
3	Oceania	5.33	6.12	13.73	74.82	LQ (1.0)
4	Native parthenogenetic	26.54	51.48	5.57	16.41	LQ (0.5)

**Table 2:** AUC, correlation, corrected AIC, delta (Δ) Akaike, and the number of parameters for each model predicting North America’s habitat suitability using the predictors from Rochlin (2018) (models 1–4) and WorldClim and ENVIREM (models 5–6). The two best models are highlighted.

Model	Model on	AUC	Correlation	Parameters	AICc	ΔΑΙCc
1	Entire range	0.95	0.64	11	2420.7	17.5
2	East Asia	0.97	0.68	8	2403.2	0
3	Oceania	0.87	0.16	9	2662.3	259
4	Parthenogenetic	0.75	0.22	8	2631.3	228
5	East Asia	0.95	0.62	8	2421.6	18.3
6	East Asia	0.96	0.64	8	2418.7	15.5

**Table 3:** Permutation importance of different environmental predictors used for MaxEnt modeling applied to the East Asia zone of *H. longicornis* occurrences.

Model 5		Model 6	
**GEZ**	45.44	**GEZ**	43.01
**bio 1**	27.72	**bio 10**	10.63
**continentality**	26.84	**bio 11**	46.36

## Supplementary Materials

The following are available online at https://www.mdpi.com/1660-4601/17/21/8285/s1. Figure S1: Frequency of Observed Presence (FOP) plots for the predictors of interest (WorldClim bio1—annual mean temperature; bio5—max temperature of warmest month; bio12—annual precipitation; bio10—mean temperature of warmest month; and bio11—mean temperature of coldest month (temperature in °C*10 and precipitation in mm); GEZ—global ecozone; ENVIREM continentality (in °C) for the East Asia region with 325 presences and 980 background points. These plots were created via the R package *MIAmaxent* [38], where the dots are the values of the predictors at the given locations, the red line a smoother regression line, and the background distribution approximate the data density; Table S1: Results of estimating the best MaxEnt model features and regularization (rm) for each geographic area of interest using the ENMeval R package [39]. Results are based on the random 5-fold method for data partitioning, where background points were randomly selected from the area of unsuitable habitat modeled from the BIOCLIM algorithm (a classic presence-only climate envelope model), and settings that primarily minimize AICc (i.e., ΔAIcc = 0) were selected for our best models (in bold); however, the AUC metrics and OR (threshold-based omission rates for test localities) metrics were calculated to select less complex models (when compared to Frequency of Observed Presence plots) and lowest number of parameters if those were giving similar or higher AUC and lowest OR because a low OR indicates less overfitting. [L: linear, Q: Quadratics, H: hinge]; Table S2: Results of the nested MaxEnt-type models built during the forward DV selection using the MIAmaxent R package [38], where DV is the derived variables from the original ones using a specified transformation [Linear, Quadratics, Monotonous, Forward or Reverse Hinge, or Threshold for Continuous variable and binary for Categorical variable] that balance complexity of model with its fitness. Alpha = 0.005 was used to set the threshold for the amount of variation a DV must explain to be kept, i.e., *P* < alpha. The selected original variables (highlighted in grey) were then entered into the ENMeval algorithm to find the best model features and regularization. The response curves for both MIAmaxent and ENMeval algorithms were compared to their corresponding Frequency of Observed Presence plot for a quick assessment.
