# Supplementary material for: Comparison of Habitat Suitability Models for Haemaphysalis longicornis Neumann in North America to Determine Its Potential Geographic Range
Source: Int J Environ Res Public Health. 2020 Nov 9;17(21):8285. doi: 10.3390/ijerph17218285 (PMC7665130; doi:10.3390/ijerph17218285)
Supplement: Supplementary file 1 [file ijerph-17-08285-s001.pdf]

Supplementary Materials

Table S1. Results of estimating the best MaxEnt model features and regularization (rm) for each geographic area of interest using the ENMeval R package [39]. Results are based on the random 5-fold method for data partitioning, where background points were randomly selected from the area of unsuitable habitat modeled from the BIOCLIM algorithm (a classic presence-only climate envelope model), and settings that primarily minimize AICc (i.e., ΔAIcc = 0) were selected for our best models (in bold); however, the AUC metrics and OR (threshold-based omission rates for test localities) metrics were calculated to select less complex models (when compared to Frequency of Observed Presence plots) and lowest number of parameters if those were giving similar or higher AUC and lowest OR because a low OR indicates less overfitting. [L: linear, Q: Quadratics, H: hinge].

Data From	*n*	Features	rm	AUC_{test}	AUC_{diff}	OR_{MTP}	OR_{10pct}	ΔAICc	Parameters
Entire distribution	407	**LQ**	**4**	**0.9758**	**0.0028**	**0.0049**	**0.1056**	**0.00**	**11**
		LQ	3.5	0.9770	0.0026	0.0049	0.1007	45.49	11
		LQ	3	0.9779	0.0026	0.0049	0.0957	161.33	12
		LQ	2.5	0.9785	0.0027	0.0049	0.1055	264.63	11
		LQ	2	0.9775	0.0032	0.0049	0.0957	289.17	12
		LQ	1.5	0.9764	0.0042	0.0049	0.1031	624.98	11
		LQ	1	0.9745	0.0048	0.0049	0.0858	986.53	13
		LQ	0.5	0.9702	0.0032	0.0049	0.1080	1614.82	14
		LQH	0.5	0.9553	0.0104	0.0000	0.1082	NA	110
		LQH	1	0.9560	0.0071	0.0000	0.1081	NA	113
		LQH	1.5	0.9538	0.0060	0.0000	0.1081	NA	105
		LQH	2	0.9565	0.0047	0.0000	0.1081	NA	110
		LQH	2.5	0.9575	0.0031	0.0000	0.1081	NA	108
		LQH	3	0.9577	0.0024	0.0000	0.1057	NA	111
		LQH	3.5	0.9622	0.0022	0.0000	0.1057	NA	94
		LQH	4	0.9642	0.0031	0.0000	0.1082	NA	113
East Asia	329	LQH	2	0.7871	0.0106	0.0061	0.1093	0.00	15
		LQH	2.5	0.7881	0.0094	0.0061	0.1063	7.46	13
		LQH	3	0.7878	0.0077	0.0030	0.0972	15.51	11
		LQH	3.5	0.7868	0.0069	0.0061	0.0941	20.27	10
		LQH	4	0.7848	0.0070	0.0061	0.0941	22.12	8
		**LQ**	**0.5**	**0.7736**	**0.0103**	**0.0061**	**0.1095**	**26.11**	**8**

		LQ	1	0.7738	0.0111	0.0030	0.1155	31.86	7
		LQ	1.5	0.7734	0.0121	0.0030	0.1155	39.82	7
		LQ	2	0.7731	0.0126	0.0030	0.1155	48.03	7
		LQ	2.5	0.7730	0.0126	0.0030	0.1124	56.28	7
		LQ	3	0.7724	0.0126	0.0030	0.1064	64.60	7
		LQ	3.5	0.7719	0.0122	0.0030	0.1064	73.15	7
		LQ	4	0.7712	0.0119	0.0030	0.1003	76.37	6
		LQH	1.5	0.7872	0.0126	0.0091	0.1124	160.73	24
		LQH	1	0.7274	0.0139	0.0121	0.0881	594.83	19
		LQH	0.5	0.5619	0.0033	0.0030	0.0212	NA	43
Native parthenogenetic	84	LQ	3	0.6635	0.0803	0.0118	0.1897	0.00	4
		LQ	2.5	0.6696	0.0790	0.0118	0.1897	0.99	5
		**LQ**	**0.5**	**0.6871**	**0.0735**	**0.0471**	**0.1544**	**1.49**	**8**
		LQ	3.5	0.6585	0.0820	0.0118	0.1779	1.53	4
		LQH	2.5	0.6741	0.0846	0.0118	0.1787	2.17	8
		LQ	1	0.6841	0.0729	0.0471	0.1426	2.38	8
		LQH	3	0.6692	0.0848	0.0118	0.1787	2.54	7
		LQ	4	0.6545	0.0804	0.0118	0.1779	3.23	4
		LQ	1.5	0.6804	0.0736	0.0235	0.1787	4.13	8
		LQ	2	0.6744	0.0768	0.0118	0.1904	4.24	7
		LQH	3.5	0.6683	0.0817	0.0118	0.1544	4.90	7
		LQH	4	0.6660	0.0767	0.0118	0.1662	5.16	6
		LQH	2	0.6814	0.0825	0.0118	0.1787	7.60	12
		LQH	1.5	0.6874	0.0871	0.0493	0.1787	13.61	17
		LQH	1	0.6891	0.0955	0.0368	0.1794	101.64	24
		LQH	0.5	0.5177	0.0602	0.0353	0.0824	NA	37
Oceania	78	**LQ**	**1**	**0.9689**	**0.0060**	**0.0250**	**0.1783**	**0.00**	**9**
		LQ	0.5	0.9704	0.0063	0.0250	0.1517	0.70	12
		LQH	3.5	0.9717	0.0060	0.0125	0.1792	4.30	11
		LQH	2	0.9724	0.0063	0.0125	0.1800	6.06	16
		LQH	3	0.9719	0.0062	0.0125	0.1792	11.74	15
		LQH	4	0.9714	0.0059	0.0125	0.1667	13.38	13
		LQ	1.5	0.9660	0.0069	0.0250	0.1792	13.52	9

LQH	2.5	0.9724	0.0061	0.0125	0.1792	14.02	17
LQH	1.5	0.9725	0.0067	0.0125	0.1800	19.16	21
LQ	2	0.9642	0.0069	0.0250	0.1792	24.98	9
LQ	2.5	0.9634	0.0069	0.0250	0.1917	32.62	7
LQ	3	0.9639	0.0064	0.0250	0.1650	41.06	7
LQH	1	0.9726	0.0071	0.0250	0.1675	43.31	26
LQ	3.5	0.9640	0.0063	0.0250	0.1525	49.92	7
LQ	4	0.9642	0.0061	0.0250	0.1525	56.36	6
LQH	0.5	0.9675	0.0122	0.0258	0.1775	197.22	37

Table S2. Results of the nested MaxEnt-type models built during the forward DV selection using the MIAmaxent R package [38], where DV is the derived variables from the original ones using a specified transformation [Linear, Quadratics, Monotonous, Forward or Reverse Hinge, or Threshold for Continuous variable and binary for Categorical variable] that balance complexity of model with its fitness. Alpha = 0.005 was used to set the threshold for the amount of variation a DV must explain to be kept, i.e., P < alpha. The selected original variables (highlighted in grey) were then entered into the ENMeval algorithm to find the best model features and regularization. The response curves for both MIAmaxent and ENMeval algoirthms were compared to their corresponding Frequency of Observed Presence plot for a quick assessment.

Round	GEZ and WorldClim Variables	m	D^2	Chi^2	df	P
1	bio11	1	0.041	187.35	1	$1.2X10^{-42}$
1	bio1	1	0.036	162.72	1	$2.9X10^{-37}$
1	bio9	2	0.036	162.19	2	$6.0X10^{-36}$
1	GEZ	2	0.030	137.89	2	$1.1X10^{-30}$
1	bio12	1	0.026	117.49	1	$2.2X10^{-27}$
1	bio16	2	0.022	101.30	2	$1.0X10^{-22}$
1	bio13	1	0.019	85.95	1	$1.9X10^{-20}$
1	bio2	1	0.017	79.02	1	$6.2X10^{-19}$
1	bio10	1	0.013	57.79	1	$2.9X10^{-14}$
1	bio14	1	0.011	48.21	1	$3.8X10^{-12}$
1	bio17	1	0.010	46.26	1	$1.0X10^{-11}$
1	bio8	1	0.009	41.29	1	$1.3X10^{-10}$
1	bio5	1	0.008	36.94	1	$1.2X10^{-09}$
2	bio11 + GEZ	3	0.053	53.93	2	$2.0X10^{-12}$
2	bio11 + bio12	2	0.051	44.47	1	$2.6X10^{-11}$
2	bio11 + bio13	2	0.049	35.95	1	$2.0X10^{-09}$
2	bio11 + bio16	3	0.049	37.23	2	$8.2X10^{-09}$

2	bio11 + bio2	2	0.046	22.60	1	$2.0X10^{-06}$
2	bio11 + bio14	2	0.045	19.02	1	$1.3X10^{-05}$
2	bio11 + bio17	2	0.045	19.02	1	$1.3X10^{-05}$
2	bio11 + bio10	2	0.044	11.10	1	$8.7X10^{-04}$
2	bio11 + bio8	2	0.044	10.83	1	0.001
2	bio11 + bio9	3	0.043	9.97	2	0.007
2	bio11 + bio5	2	0.043	7.05	1	0.008
2	bio11 + bio1	2	0.041	0.41	1	0.521
3	bio11 + GEZ + bio10	4	0.055	10.32	1	0.001
3	bio11 + GEZ + bio8	4	0.055	8.91	1	0.003
3	bio11 + GEZ + bio12	4	0.054	4.23	1	0.040
3	bio11 + GEZ + bio2	4	0.054	4.05	1	0.044
3	bio11 + GEZ + bio14	4	0.054	3.56	1	0.059
3	bio11 + GEZ + bio17	4	0.054	3.47	1	0.063
3	bio11 + GEZ + bio13	4	0.054	2.79	1	0.095
3	bio11 + GEZ + bio16	5	0.054	3.16	2	0.206
4	bio11 + GEZ + bio10 + bio8	5	0.056	1.52	1	0.218
5	bio11 + GEZ+ bio10 + bio10:bio11	5	0.056	3.06	1	0.080
5	bio11 + GEZ + bio10 + bio11:GEZ	6	0.055	0.00	2	1.000
5	bio11 + GEZ + bio10 + GEZ:bio10	6	0.055	0.00	2	1.000

round	GEZ, WorldClim and ENVIREM variables	m	Dsq	Chisq	df	P
1	Continentality	1	0.044	198.36	1	$4.8X10^{-45}$
1	bio11	1	0.041	187.35	1	$1.2X10^{-42}$
1	bio1	1	0.036	162.72	1	$2.9X10^{-37}$
1	bio9	2	0.036	162.19	2	$6.0X10^{-36}$
1	GEZ	2	0.030	137.89	2	$1.1X10^{-30}$
1	bio12	1	0.026	117.49	1	$2.2X10^{-27}$
1	bio16	2	0.022	101.30	2	$1.0X10^{-22}$
1	bio13	1	0.019	85.95	1	$1.9X10^{-20}$
1	bio2	1	0.017	79.02	1	$6.2X10^{-19}$
1	aridityIndex	1	0.015	67.31	1	$2.3X10^{-16}$
1	annualPET	2	0.015	69.85	2	$6.8X10^{-16}$
1	bio10	1	0.013	57.79	1	$2.9X10^{-14}$
1	bio14	1	0.011	48.21	1	$3.8X10^{-12}$

1	bio17	1	0.010	46.26	1	$1.0X10^{-11}$
1	bio8	1	0.009	41.29	1	$1.3X10^{-10}$
1	bio5	1	0.008	36.94	1	$1.2X10^{-09}$
2	continentality + GEZ	3	0.056	57.72	2	$2.9X10^{-13}$
2	continentality + bio12	2	0.055	51.37	1	$7.6X10^{-13}$
2	continentality + bio13	2	0.053	42.05	1	$8.9X10^{-11}$
2	continentality + bio16	3	0.054	46.26	2	$9.0X10^{-11}$
2	continentality + aridityIndex	2	0.050	29.93	1	$4.5X10^{-08}$
2	continentality + bio2	2	0.048	21.34	1	$3.8X10^{-06}$
2	continentality + bio14	2	0.048	18.30	1	$1.9X10^{-05}$
2	continentality + bio17	2	0.048	18.05	1	$2.2X10^{-05}$
2	continentality + bio1	2	0.048	17.70	1	$2.6X10^{-05}$
2	continentality + bio10	2	0.047	13.63	1	0.0002
2	continentality + bio8	2	0.046	12.97	1	0.0003
2	continentality + bio11	2	0.046	11.89	1	0.0006
2	continentality + bio9	3	0.046	10.95	2	0.004
2	continentality + bio5	2	0.045	6.25	1	0.012
2	continentality + annualPET	3	0.044	1.68	2	0.431
3	continentality + GEZ + bio1	4	0.059	12.32	1	0.0004
3	continentality + GEZ + bio10	4	0.058	9.74	1	0.002
3	continentality + GEZ + bio8	4	0.058	7.40	1	0.007
3	continentality + GEZ + bio12	4	0.058	6.46	1	0.011
3	continentality + GEZ + aridityIndex	4	0.057	5.28	1	0.022
3	continentality + GEZ + bio11	4	0.057	4.96	1	0.026
3	continentality + GEZ + bio13	4	0.057	4.66	1	0.031
3	continentality + GEZ + bio16	5	0.058	6.86	2	0.032
3	continentality + GEZ + bio14	4	0.057	3.15	1	0.076
3	continentality + GEZ + bio2	4	0.057	3.01	1	0.083
3	continentality + GEZ + bio17	4	0.057	2.94	1	0.087
3	continentality + GEZ + bio9	5	0.057	1.05	2	0.591
4	continentality + GEZ + bio1 + bio10	5	0.059	1.98	1	0.160
5	continentality + GEZ + bio1 + continentality:bio1	5	0.059	0.35	1	0.554
5	continentality + GEZ + bio1 + continentality:GEZ	6	0.059	0.00	2	1.000
5	continentality + GEZ + bio1 + GEZ:bio1	6	0.059	0.00	2	1.000

m: number of DVs in a model, D^2: a fraction of deviance explained, Chi2: Chi-squared statistic for the model nested comparison, df: a degree of freedom, P: p-value of the Chi^2 statistic under the specified df.

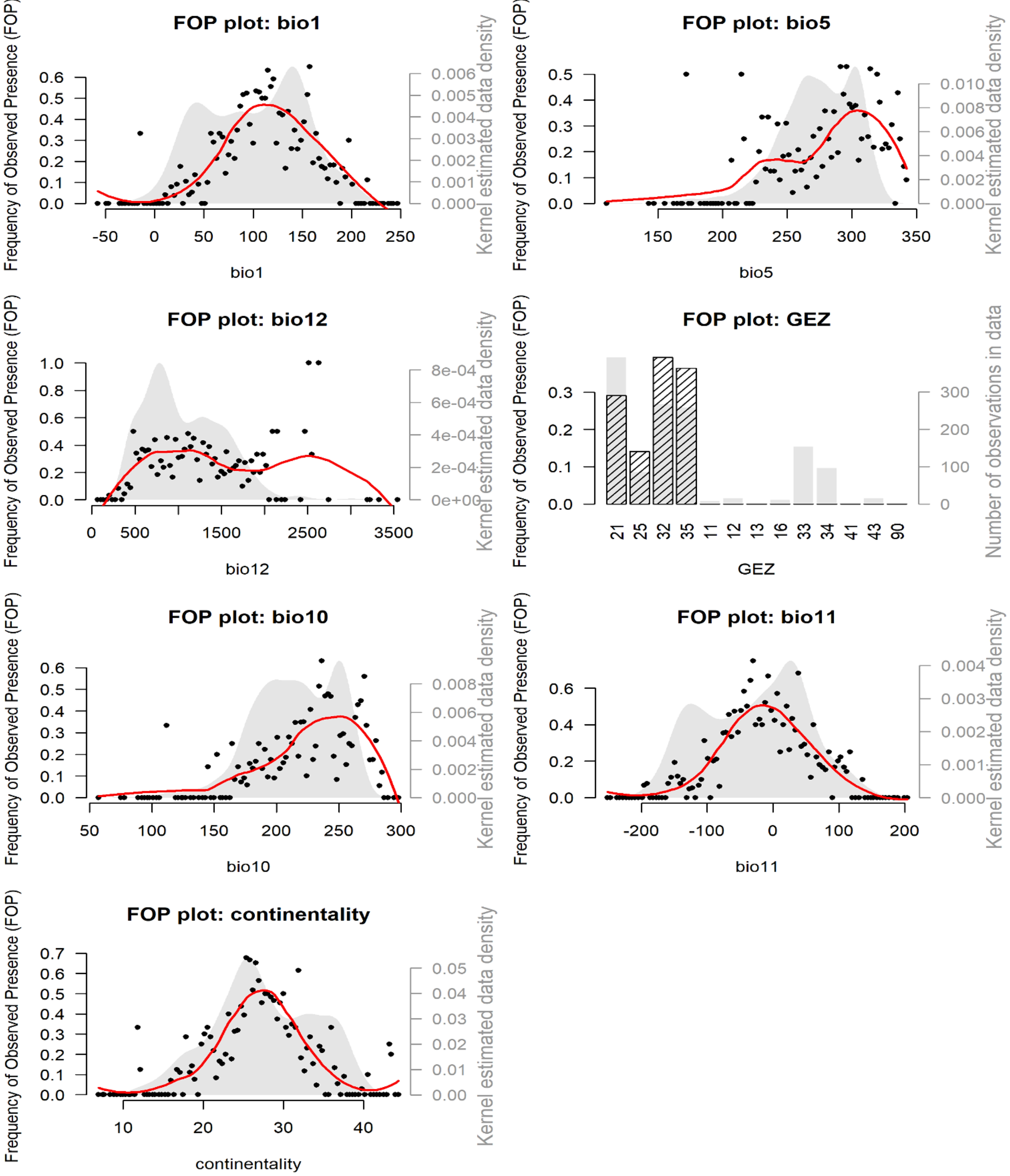

Figure S1. Frequency of Observed Presence (FOP) plots for the predictors of interest (WorldClim bio1—annual mean temperature; bio5—max temperature of warmest month; bio12—annual precipitation; bio10—mean temperature of warmest month; and bio11—mean temperature of coldest month (temperature in °C*10 and precipitation in mm); GEZ—global ecozone; ENVIREM continentality (in °C) for the East Asia region with 325 presences and 980 background points. These plots were created via the R package *MIAmaxent* [38], where the dots are the values of the predictors at the given locations, the red line a smoother regression line, and the background distribution approximate the data density.
